# The physician’s role and empathy – a qualitative study of third year medical students

**DOI:** 10.1186/1472-6920-14-165

**Published:** 2014-08-09

**Authors:** Hanne-Lise Eikeland, Knut Ørnes, Arnstein Finset, Reidar Pedersen

**Affiliations:** 1 Department of Behavioral Sciences, Institute of Basic Medical Sciences, University of Oslo, Domus Medica, Sognsvannsveien 9, Oslo 0372, Norway; 2 Institute of Health and Society, Centre for Medical Ethics, University of Oslo, Kirkeveien 166, Fredrik Holsts hus, Oslo 0450, Norway

**Keywords:** Empathy, Medical education, Professionalism

## Abstract

**Background:**

Empathy is important in ensuring the quality of the patient-physician relationship. Several studies have concluded that empathy declines during medical training, especially during the third year. However, there is little empirical research on what may influence a medical student’s empathy. In addition, studies of empathy in medicine have generally been dominated by quantitative approaches, primarily self-assessment questionnaires. This is a paradox given the complexity and importance of empathy. In this paper we explore medical students’ opinions of what may foster or inhibit empathy during medical school, with a particular emphasis on how empathy is influenced by the initiation into the physician’s role.

**Methods:**

We performed semi-structured qualitative interviews with 11 third year medical students. Content analysis was used to analyse the transcribed interviews.

**Results:**

Five aspects of the the physician’s role and the students’ role acquisition emerged when the students were asked to describe what may influence their empathy: 1) Becoming and being a professional, 2) Rules concerning emotions and care, 3) Emotional control, 4) The primary importance of biomedical knowledge, and 5) Cynicism as a coping strategy.

**Conclusion:**

This study suggest that the described inhibitors of empathy may originate in the hidden curriculum and reinforce each other, creating a greater distance between the physician and the patient, and possibly resulting in decreased empathy. Mastering biomedical knowledge is an important part of the students’ ideals of the physician’s role, and sometimes objective and distanced ideals may suppress empathy and the students’ own emotions.

## Background

Empathy is of utmost importance for any physician, and is a necessary condition in order for the physician to understand the individual patient’s needs and experiences. Empathy has been defined and studied in various ways and neither a “gold standard” method nor a unified definition exists. Definitions of empathy vary, and may include cognitive, emotional, behavioural, interpretive, and moral aspects [1,2]. In order to include all these aspects we define empathy in medicine as an appropriate understanding and communication of the patient’s experiences [2]. Empathic physicians can encourage a patient’s feelings of safety and trust in the care giver, facilitate disclosure of key information and improve patient satisfaction and compliance [3]. Empathy has also been shown to improve the therapeutic effect and the patient’s quality of life [3-7]. The empathic physician may profit from increased diagnostic accuracy, more meaningful work, an increased sense of well-being, and reduced symptoms of burnout [3,4,8-12].

A recent review of empathy development concluded that empathy declines during medical training [13]. The importance of these changes have been debated [14] and more recent studies show different trends in medical students’ empathy development [15-23]. Although hotly debated [6,24-26], there is sparse empirical research on what may influence medical students’ empathy [13,27].

In general, the study of empathy in medicine has been largely dominated by quantitative self-assessment approaches [28], while qualitative methods have rarely been used. This is a paradox, considering the importance and complexity of the concept of empathy.

The aim of this study was to better understand what may foster or inhibit medical students’ empathy. We performed a qualitative study, and the research questions were the following:

1. How do medical students perceive the significance of empathy?

2. What do medical students believe promotes and/or inhibits their ability to empathise with patients.

## Methods

We recruited 11 third year medical students at the University of Oslo, Norway, in their first year of clinical training, since this is regarded as a crucial period for empathy development. The medical education in Oslo is 6 years long. The first 2 years teaches the students human biology, biochemistry and physiology. The third year is the first year with clinical training and in this year the students interact with patients several times a week. See “Medical Education” subsection for more details on the medical education in Oslo.

All the recruited students had completed mandatory courses in communication skills as part of the medical training at the University of Oslo. The students undertook rotations in internal medicine and surgery during which they practiced patient interactions alone, in small groups and together with a medical doctor. To increase the variation, students were recruited from two different classes. After a lecture, potential participants received information about the study. Students who were interested signed up on a list, in total 19 students. Eleven students six female and five male were interviewed. Thus, only 11 of the initial 19 of the students were included in the study as the richness of the data was then sufficient to answer the research questions.

The students were interviewed between the spring and autumn of 2011, using a semi-structured interview guide. The interviewers were two of the authors, HLE and KØ, both 5^th^ year medical students at the time of data collection.

Written, voluntary, and informed consent was obtained from all participants. Since the study was not defined by the Regional Ethics Committee as health research, the study was exempted from the requirement of study pre-approval.

The interviews lasted 60–80 minutes and were transcribed verbatim. The students were thoroughly informed that the interview was not an examination but an attempt to share thoughts and experiences. The students were asked questions exploring their views on empathy and its influences. If they provided answers that were vague or needed more detailed reflection, follow up questions were asked. In the interview guide questions are presented in full sentences but in reality the formulation, as well as the order of the questions, were adjusted according to the situation. Both the interviewers (HLE, KØ) were at that time medical students with their own experiences that might influence the interviews and the interpretation process. This influence may have had both a positive and a negative impact. On the one hand, having shared experiences might have made closer connection with the participating students. On the other hand, the interviewers might have had similar pre-suppositions as the participants, something which may sometimes inhibit making the implicit more explicit.

The data was approached in an interactive process. Data analysis started early in the data collection process. The authors regularly shared experiences and this constituted a basis for individual and joint reflection and discussion. We performed a qualitative content analysis of the data [29,30] based upon themes emerging from the texts, relevant theories, and earlier research. After reading through the interviews several times to get a sense of meaning, and discussing our preliminary analyses, we saw that the physician role and its relation to empathy emerged as an important overarching theme in answering research question 2 above. The physician role was not mentioned in the interview guide or by the interviewers. Two of the authors proceeded by reading and analysing the interviews more thoroughly (HLE and RP). Further discussions of meaning were had before all interviews were analysed according to a list of themes. Passages pertaining to the various themes were then selected and condensed by two of the authors (HLE and RP). Below, these themes are presented through condensed text and illustrative quotes. The qualitative component of our study adheres to the RATS guidelines for reporting qualitative studies.

### Medical education in Oslo

– 1st year: Human biology, Communication, Society and Method, Cell Biology

– 2nd year: Organ systems, Nutrition, Musculoskeletal system

– 3rd year: Circulation, Respiration, Nephrology, Nutrition, Hematology, Dermatology

– 4th year: Neurology, Ear-Nose-Throat, Ophthalmology, Psychiatry

– 5th year: Reproduction, Obstetrics, Gynecology, Pediatrics. Health and Society. Student are deployed for 3 months at a General Practitioners office and at a Hospital.

– 6^th^ year: Clinical Medicine

## Results

All of the students described how their understanding of the physician’s role and their role acquisition may influence their empathy. Five different aspects emerged. These will be presented separately below, although the students’ answers indicate that they are often closely related and may overlap.

### Becoming and being a professional

All students emphasised the need to be a professional when talking to a patient, and described various norms, ideals and strategies that are important in becoming a professional. Developing a certain emotional distance from the patient, and avoiding too much empathy was widely understood as being a key component of being a professional. One student was very conscious that she should not be a friend, or behave as a family member, but instead create a professional distance. The student vigorously tried to create distance, and avoided thoughts like “What if it was me, or my sister”.

#### How to balance between distance and empathy?

Several students were of the opinion that it is important to find the correct balance between distance and empathy towards patients. However, they were uncertain where to draw the line. Descriptions of distance differed, some of the students found it to be positive while others were more ambivalent.

One student stated that distance is required in order to function in a professional role and to be clearheaded. A different student was concerned that too much distance can make it difficult to relate to the patient’s situation. This student reflected on a prior experience in the emergency department where a patient with acute pancreatitis waited an hour before being given pain relief*.*

Student: I was in the Emergency Department (ED) yesterday (…)a patient who had acute pancreatitis was in severe pain and the nurses said “The patient needs painkillers now.” When I informed the doctor, he said, “Yes, yes, I’m coming”. It took an hour before he came, I know it is in the ED and that they need to prioritise,(…)I think (…) in a way you should try to maintain it, that is to say, that when you see things for the first time, you react entirely differently than what you do when you have done the same thing ten thousand times - but one way or the other trying to keep that first thought (…) it is extremely difficult, but the patients could probably benefit if you were a bit more humble in relation to things like this.

Interviewer 1: (…) what does an episode like that result in?

Student: it probably results in that the next time a patient is lying there in pain, I will probably become more aloof. I may think that it is not that bad, the patient will survive, the doctor will come as soon as he can and there is nothing more I can do. (…) like yesterday, I had palpitations (…) next time maybe I won’t get palpitations and I won’t be that stressed.

#### Vulnerable or immortal and callous?

In contrast to these views on professional distance, some students used their own life experiences in order to actively foster empathy towards their patients. One student brought up how prior experiences, in particular those concerning feelings of vulnerability and mortality, would enhance empathy for the patients. According to some of the students, a medical education might condition students to view themselves as immortal. Furthermore, they thought that one will invariably become emotionally blunted the longer one works as a doctor. Some students found it challenging to be humane and a professional at the same time, and they often described being a professional as an antithesis to being humane. Reconciliation of emotions and reason was a struggle for the students. One student argued that it must be possible to be empathic without being seen as a failed doctor and without spending your spare time thinking about patients.

### Rules for emotions and care

There was a strong conviction among the students that there are rules regulating the display of emotions towards patients. Some students referred to rules that they were taught, e.g. that crying is not allowed during a consultation because this is something that you cannot control. Another student felt that her medical education compelled her to show emotions towards patients differently than towards others in non-professional settings. Consequently, she now both thought, and categorised her thoughts, in a different way, and suppressed her emotions when interacting with patients.

One student explained that exploring a patient’s true feelings is not permitted. She noted that she explored and discussed patients’ emotions less than before because she felt that it is not accepted within the medical educational environment.

It is more natural for us to ask about passing urine and stools than it is to ask whether the person is feeling well… and actually dare to say things like “Are you sure that you are feeling well? … To take that step, It is not acceptable to double check that the person spoke truly about his or her own feelings.

This student also discussed a particular incident where her fellow student held the hand of a mentally challenged patient during a consultation. Afterwards, the teacher told the student that this was not allowed. In general, the students learned these rules through clinical training – i.e. explicit or implicit rules conveyed by senior doctors. In one student’s opinion, the doctors had their own personal style that they expected the students to adapt to. This student wanted to demonstrate empathy towards the patients in ways that she was not allowed, for example by attending and responding to care needs.

there are some of the elderly patients (…) that ask if someone can cut their nails (…) My idea of empathy is that that is something I could have done, (…) I changed the bedding for a patient who had vomited (…) then I was told, “Then you should call the nurse because she’s supposed to do that,” because we were seven people, we were supposed to interview (…) and I thought, “Well, there’s only two people talking. I can do this in the meantime”.

### Emotional control

Being able to control emotional reactions towards patients was regarded by some of the students as being an important part/element of a professional behaviour. Controlling emotions had a close link to distance for the students, as it involved suppressing or postponing emotional reactions during the interaction.

Some students felt that now, in their third year of medical studies, they felt more in control of their emotions. One student felt that now she could approach the patient with a certain “professional distance”, and that she interacts with patients in a different way now than in the beginning of her studies. She adopts a professional role when she dons the doctor’s coat/wears the white coat. Another student described how she now possesses greater self-control and comfort when interacting with people that she doesn’t have a close relationship with.

The students differed in how they described their emotional control. Some used the term “to put emotions on hold.” One student reflected upon her own emotional reaction after attending a nightshift where a patient suddenly died after a blood-pressure fall. After the incident the doctors systematically evaluated the medical procedures and, at the time, the student found this satisfying. Her emotional reaction to the death (weeping) occurred the morning following the nightshift. Her ability to delay her reaction until after the shift was over gave this student satisfaction. She perceived this as having demonstrated the professional behaviour that is expected from health-personnel, and accentuated how important it is to have control over your own emotional reactions in order to be a professional.

Many of the students reflected on the fact that their emotions and emotional reactions have changed during the course of their medical education. One student experienced guilt when after having spoken with terminal patients he became aware that he didn’t exhibit any emotional response. He felt that it is a virtue to meet patients with an adequate level of empathy and compassion, but at the same time that it is easy to become distanced from one’s patients. Several students were comfortable with this distance, but some wondered how other people will perceive this change.

#### Avoiding emotional conflict and disharmony through distance

A number of students have had negative reactions towards patients. Some of the students experienced conflict when they encounter patients with views that they disagree with. They were afraid to show empathy towards these patients because it might be interpreted as if they agree with, or even like, the patient. One of the students felt irritated if patients treated him badly, or criticised him or his colleagues; and he found it hard to demonstrate empathy when he disagreed with or disliked the patient. Another student thought it was easier to communicate with patients with whom he did not have a good rapport because then he was not afraid to hurt the patient’s feelings, and thus became less nervous during the interaction.

### The primary importance of biomedical knowledge

The students shared the opinion that possessing biomedical knowledge takes precedence over their ability to manage the emotional aspects of the consultation. Some students also emphasised that emotions distract them from clear, rational thinking, and therefore impaired their ability to make sound professional decisions. According to one student, distance between the physician and the patient is important in developing a professional character, and being able to give sound, objective advice. Another student wanted to be both academically skilled and empathetic, but felt that being bio-medically skilful is given higher priority throughout medical school. In his opinion, one learns only to diagnose, refer patients, and relate to a time schedule during medical school. “… We should be (…) academically strong, but we should then, in a way, be humane as little as possible”.

Many of the students acknowledged the importance of empathy and communication in patient interactions, but questioned the likelihood that they will have sufficient time to make use of this kind of competence and ideal. One student explained how they learn a lot about behavioural science, communication, and empathy, but questioned whether there is enough time to do things the “right” way. He experienced in clinical tutoring, training in small groups where the physician teacher interact with a patient in front of or together with the students, that patients were interrupted because of time constraints, and that the focus was on taking their medical history and conducting the physical examination. The student found this difficult to deal with.

#### Cognitive overload when attempting to “do both”

A number of students found it hard to incorporate both the biomedical and the communicative aspects into consultations. For example, one student explained that when he is focusing on communicative aspects, he might forget to ask the most important biomedical questions, e.g. diagnostic questions about bodily functions. He found it hard to handle communication skills and empathic relationships with patients because it might compromise his biomedical knowledge. His experience was that one cannot use or integrate both of these perspectives concurrently. To date, he had prioritized demonstrating empathy towards the patients, and felt that this may have affected his diagnostic reasoning negatively.

### Cynicism as a coping strategy

The students used diverse terms to describe the need to develop an emotional distance; e.g. as a way to cope or survive. The word cynical was used by many of the students. Most of the students described cynicism as a positive virtue, or an accepted coping strategy, rather than a development to be concerned about. One student explained how she needs cynicism because it provides her with emotional control, and she felt that this was a consequence of being exposed to many tough situations. This student reflected upon how she sometimes forgot that the patients were real humans and not just opportunities for learning about medicine. She felt an incipient cynicism and felt more cynical this year than before, and was more conscious and selective about what she allowed herself to be affected by. She thought it was important to reflect upon this in order to develop empathy.

I also think that as a student you can become a bit cynical (…) when you have in bedside teaching (…) and you listen and you auscultate back No. 8, it is no longer humans (…) with names, but it’s more back No. 8. So I think you can quickly become cynical if you are not aware. When you are sitting in the Emergency Department and hoping for an acute myocardial infarction and trauma (…), I think you can quickly become cynical.

Another student thought that you might become cynical when the frame of reference used to evaluate other people becomes influenced by your own cumulative experiences. You forget what is perceived by the wider community as normal. One student felt that she thinks less about the patient’s feelings than before and that this might be a direct consequence of being a medical student. Yet another student had the perception that doctors, in general, are less empathetic, and that they may become emotionally callous to avoid being affected by their patients and to get through their everyday life as a doctor.

## Discussion

The interviewed medical students emphasise that throughout medical school academic skills are prioritized over humanistic knowledge, and that this is an important part of their understanding of the physician’s role. In today’s medical education and practice, there is a strong emphasis on evidence based medicine and biomedical knowledge. Although “softer” aspects, such as professionalism, developing the physician’s role, and empathy, are often highlighted as important, these aspects are most often given lower priority and primarily conveyed through the informal and hidden curriculum. Hidden curriculum is defined as influences at the level of organizational structure and culture, referring to processes that are often unarticulated and unexplored [31].

The findings in this study indicate that time constraints make the students leave out what is not top priority. Furthermore, the priority given to various types of knowledge and skills in medical school, may influence how medical students develop their role understanding and define their responsibilities as professionals. These factors may possibly also affect their empathic skills.

Medical students experience a socialization process in medical school, participate in role-defining situations and acquire new norms and ideals. They are confronted with some of the most difficult questions – e.g. how to deal with life and death, and how to handle their own and the patient’s emotions in very demanding situations. At the same time they are expected to acquire and apply large amounts of new knowledge and skills. These transformations may separate the medical student from the general public [31] and also alienate the students from their own emotions and existential identity. Several authors have assumed that medical education increases distress among students, and that this in turn leads to a decline in empathy [9,13,32-35]. This study gives some empirical support to these assumptions and the participating students also describe why this may happen, both through the so-called hidden curriculum [36] and as an unintended side-effect of the more formal parts of medical training.

The role acquisition processes, and the accompanying ideals and strategies depicted in the interviews, are mostly inhibitors, rather than promoters of empathy. This study was not designed to study the possible relations or hierarchy between these possible influences of medical student’s empathy. However, the described focus on biomedical knowledge, rules, control, suppression of emotions, human distance, and cynicism as morally neutral, echoes what has often been described as objectivistic ideals. One may also speculate whether some of these tendencies also undermine so-called “double-loop learning” and reflection [37]. From a philosophical hermeneutical perspective, one may argue that the various ideals, norms, strategies and expectations described by the students, creates a horizon that delimits what the students are able to perceive or understand in a clinical situation [27]. Thus the five aspects of the physician’s role-development described in this study may reinforce each other – and in sum create a greater distance both from the patient and from the students’ own emotions and reactions.

The findings suggest that the students are struggling to cope and protect themselves in an intense learning environment. Some distance may be appropriate, but with too much distance there is a danger that the physician becomes indifferent and does not manage to engage in a genuine dialogue with the patient. Since the students’ development of a specific understanding of the physician’s role, and their acclimatization to this new role, is by and large not part of the formal curriculum, there is less awareness of the topic. Thus, these transformations are likely to elude supervision, discussion and critique. The role acquisition process and the strong emphasis on facts and skills may also alienate the students from their own feelings and experiences, thus undermining the possibility for self-reflection and emotional development among the students.

The students seem to expect that the right answer or solution to how to handle clinical situations exists, or that there is always a right thing to do. Such expectations may sometimes be very unrealistic. The students’ dependence on rules may be perceived as a natural step towards professionalism as described by Dreyfus (see Table 1[38,39]). However, with little explicit focus on this in the formal curriculum, it may be less likely that the students will proceed to more advanced stages when it comes to defining and developing their professional role.

**Table 1:** Dreyfus model of skill acquisition

**Novice**	The student is rigidly adherent to rules.
**Advanced beginner**	The student is not able to discriminate between different aspects of the clinical situation.
**Competent**	The student is now able to identify all relevant elements in the clinical situation and is easily overwhelmed.
**Proficient**	The student has an holistic understanding and is able to discriminate between different aspects of the clinical situation.
**Expert**	The student has an intuitive understanding of the clinical situation based on experience.

Some of the students struggle to reconcile reason and emotions, and view their own emotions as a threat to rationality rather than applying a more integrated view. However, there are strong philosophical arguments and empirical evidence that indicate that emotions, at an appropriate level, may function as an important source of information and trigger for self-reflection, helping the physician to identify important phenomena in the consultation [11,40].

The students’ struggle to control and suppress emotions may be a result of not knowing how to handle emotions, e.g. around death, suffering, medical errors and their own mortality [41]. With increasing emotional distance, empathy towards the patient may be reduced. Moreover, there may be a connection between the suppression of feelings and the rate of burnout and suicide among physicians, which has been shown to be higher than in the general population [41,42].

Some students are pleased to gain greater distance from their emotions and even to become more cynical, because they, in this way, possess greater control of the situation. This echoes earlier research by Feudtner et al., who found that students who report erosion of their ethical principles are not displeased with their own ethical development, as if they have accepted that becoming a doctor demands a change in character and principles [43]. Research on empathy development in medical students also lend some support to the findings that medical students lose some of their humanity, and develop a more cynical attitude towards patients, referring to this as coping or survival strategies [13,25,26,32]. What our study adds is a more detailed description of how the hidden curriculum, role-expectations, and epistemological ideals may influence empathy. More generally this qualitative study presents possible explanations of the stunting or deterioration of empathy described in earlier studies in medical schools. However, further and more rigorous studies are required before we can give more trustworthy answers on what inhibits and promotes empathy in medical training.

We used a qualitative approach to gain an in-depth understanding of possible influences on empathy in medical students. Important limitations are: One, the population was a small group of students from one single institution. Two, there is a possibility that the students most motivated to communicate with patients are overrepresented in this study, this could have had influence on the results.

## Conclusion

This study suggest that the described inhibitors of empathy may originate in the hidden curriculum and reinforce each other, creating a greater distance between the physician and the patient, and possibly resulting in decreased empathy. Mastering biomedical knowledge is an important part of the students’ ideals of the physician’s role, and sometimes objective and distanced ideals may suppress empathy and the students’ own emotions.

We hope the readers will discuss whether our findings are transferable to other medical students and medical schools, and that further research is undertaken to study the possible relations between physician’s role development and empathy. The findings in our study indicate that there is a need to discuss what kind of physician’s role we want medical students to assume, in particular to foster appropriate levels of distance, control and emotional regulation, and to explore how the formal and informal curriculum may incorporate both biomedicine and empathy.

## Competing interests

All authors (HLE, KO, RP and AF) declare that they have no competing interest: no support from any organisations, no financial relationships with any organisations and no other relationships or activities that could influence the submitted work.

## Authors’ contributions

HLE developed the research design, recruited participants, interviewed students, developed the interview guide, analysed the qualitative data material and wrote the manuscript. KO developed the research design, recruited participants, interviewed students and participated in writing. AF developed the research design and participated in writing. RP developed the research design, developed the interview guide, analysed the qualitative data material and wrote the manuscript. All authors read and approved the final manuscript.

## Authors’ information

HLE: Graduate from the medical education and student researcher-program at the University in Oslo. Working as a doctor at Molde Hospital, Norway.

KO: Graduate from the medical education and student researcher-program at the University in Oslo. PhD student at the University in Oslo.

RP: MD, MA, BA, PHD. Researcher/professor at the Institute of Health and Society, Centre for Medical Ethics, University of Oslo

AF: Psychologist, PhD, Professor at the Department of Behavioral Science at the University in Oslo.

## Pre-publication history

The pre-publication history for this paper can be accessed here:

http://www.biomedcentral.com/1472-6920/14/165/prepub
